# Manufacturing of Porous Glass by Femtosecond Laser Welding

**DOI:** 10.3390/mi13050765

**Published:** 2022-05-12

**Authors:** Hua Tan, Jiahui Pan, Xiaojia Zheng, Xiaoquan Fu, Yuxun Zhang, Yanxing Liu, Qiheng Huang

**Affiliations:** 1 Dongguan University of Technology, Dongguan 523808, China; 13435625909@163.com (J.P.); zxj18718286049@163.com (X.Z.); fxq10262022@163.com (X.F.); zhangyx@dgut.edu.cn (Y.Z.); talkingbird@dgut.edu.cn (Y.L.); kopft1556346407@126.com (Q.H.); 2 State Key Laboratory of High Performance Complex Manufacturing, College of Mechanical and Electrical Engineering, Central South University, Changsha 410083, China; 3 College of Mechanical Engineering, Hunan Institute of Science and Technology, Yueyang 414000, China

**Keywords:** femtosecond laser, glass welding, manufacturing processes, porous glass, functional glass

## Abstract

Based on femtosecond laser glass welding, four different porous structures of welding spots were formed by the manufacturing processes of spatiotemporal beam shaping and alternating high repetition rate transformation. Compared with an ordinary Gaussian beam, the welding spot fabricated by the flattened Gaussian beam had smoother welding edges with little debris, and the bottom of the welding spot pore was flat. Instead of a fixed high repetition rate, periodically alternating high repetition rates were adopted, which induced multiple refractive indices in the welding spot pore. The welding spot pores manufactured by spatiotemporal beam shaping and alternating high repetition rate transformation have a special structure and excellent properties, which correspond to superior functions of porous glass.

## 1. Introduction

Due to its particular structure, porous glass has the unique advantages of a large surface area, a controllable pore diameter, stable chemical properties, and high deformation resistance; it has been widely applied in the fields of optical storage, eco-environmental protection, enzyme immobilization, virus filtering, chromatography, optical fiber communication, and so on. It is gradually becoming one of the high-tech materials in a variety of applications [1].

In the 1930s, the Hood group, the father of porous glass from Corning in the United States, employed glass of different compositions as a matrix and obtained a kind of amorphous inorganic nonmetallic material with a porous structure through the processes of phase splitting and leaching. After that, as a new type of functional material, porous glass gradually became used [2].

Currently, the traditional techniques in the manufacturing of porous glass mainly include powder sintering, phase splitting, sol-gel, etc. [1].

In the manufacturing of porous glass, powder sintering is a technique involving first blending of glass powder, aluminum powder, and copper powder together and then flowing the powder into molds after grinding, drying, and filtering. Finally, porous glass is formed into shapes from the molds through heat treatment. However, there are several major drawbacks to this method, such as higher environmental pollution due to the sintering processes, longer manufacturing periods, and higher requirements on the particle size of the base materials [3,4,5].

The method of phase splitting is used to manufacture nanoporous glass by dissolving one of the glass phases by phase-separation technology. The first step is to make a mixture of sodium carbonate, quartz sand, and borax, followed by melting it at 1500 degrees Celsius, eventually forming borosilicate glass with a porous structure. The process is primarily used for manufacturing porous glass in bar, tube, and flat shapes. However, the main defect is that the production cycle is too long [6,7,8].

In the sol-gel process, porous glass is synthesized with boric acid, ethyl silicate and sodium acetate as raw materials, water and ethyl alcohol as solvents, and hydrochloric acid as a catalyst. It often takes several days, even weeks, to achieve the end product. Meanwhile, the process is accompanied by toxic substance emission and has some effects on the accuracy of porous glass due to pore shrinkage [9,10,11].

The three porous glass manufacturing processes mentioned above have the disadvantages of chemical pollution, long periods, high costs and low precision, which have become bottlenecks restricting the entry of glass products into the high-tech application domain.

Femtosecond laser welding as a cutting-edge technology has some significant benefits, such as high precision, high speed, a noncontact character, a low damage threshold, and a small heat-affected zone. In this paper, we performed research on porous glass manufacturing by femtosecond laser welding, which involves the processes of spatiotemporal beam shaping and alternating high repetition rate transformation. The manufacturing processes not only have the advantages of one-time forming, no chemical material involved, a short production cycle, a smaller heat-affected zone, etc. but they also provide a technology for the production of porous glass with multiple refractive indices. This greatly expands the functional application prospects of porous glass in the fields of industry, agriculture, medical treatment, food products, environmental protection, etc.

## 2. Experimental Setup and Manufacturing Processes

### 2.1. Experimental Setup

**Figure 1:**
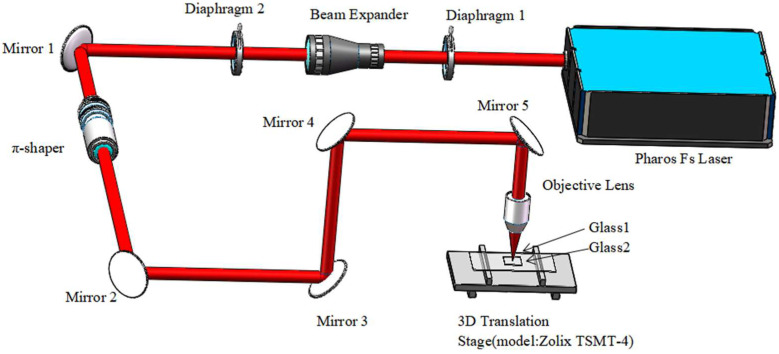
Schematic of the experimental setup.

The amplified femtosecond Pharos laser system used for manufacturing porous glass by femtosecond laser welding is sketched in Figure 1. It consisted of a Yb:KGW oscillator and a regenerative amplifier. In our experiments, the system provided pulses at a wavelength of 1030 nm, an average output power of 12 W, a 5-μm-diameter beam spot, and a pulse duration of 1000 fs. The femtosecond laser with a pulse duration of 1000 fs has lower propagation loss [12] and stronger thermal effect accumulation [13], which can facilitate the formation of color center [13] and deeper working depth [14]. We use the high repetition rate ranges from 200 kHz to 2 MHz. In order to investigate the structures of four types of welding spots, the pulse energies were set as 20 μJ, 15 μJ, and 12 μJ, which corresponded to the typical high repetition rates of 600 kHz, 800 kHz, and 1 MHz, respectively. The laser beam was focused by a 20×, 0.4 NA lens onto the interface of two glass substrates (30 mm×30 mm×1 mm, JGS2, refractive index of 1.45), which was moved by a computer-controlled 3D translation stage (model: Zolix TSMT-4). The Prism-Pro Measuring Instrument was used to measure the refractive index of glass before and after welding. Spatiotemporal beam shaping was performed by a π-shaper beam shaping lens. The welding speed is set to 2 mm/s. Because the welding surface is a square with 30 mm on each side, 15 s are needed to create a welding line in the middle of the surface. To avoid manual errors, we performed each experiment 10 times on average.

### 2.2. Manufacturing Processes

Nonlinear welding effects occurred when the femtosecond laser interacted with the interface of the two glass substrates. Porous glass was produced by the processes of spatiotemporal beam shaping and alternating high repetition rate transformation, and its pores were on the micrometer scale. On the one hand, we can form structural pores with different sizes and shapes by using spatiotemporal beam shaping. On the other hand, multiple refractive indices come into being in the weld spot pore through alternating high repetition transformation.

## 3. Mechanism

### 3.1. Comparison of a Gaussian Beam and a Flattened Gaussian Beam

**Figure 2:**
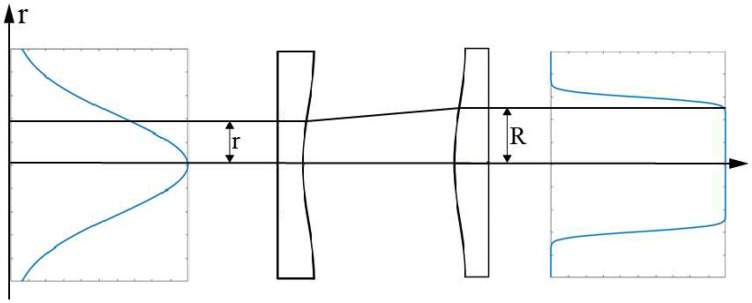
The π-shaper beam shaping configuration consists of two plano-aspheric lenses. The first surface redistributes while the second surface recollimates the rays [15].

A Gaussian beam can be converted to a flattened Gaussian beam by the use of spatiotemporal beam shaping. Figure 2 shows a π-shaper beam shaping configuration which consists of two plano-aspheric lenses transforming a Gaussian input into a flat-top output intensity distribution with a gradual roll-off from the uniform to null region. The indicated geometric ray mapping then describes where an initial parallel ray with radial distance *r* is refracted at the first aspherical surface to be redirected in parallel direction with radial distance *R* at the second aspherical surface [15].

The energy distribution of the laser spot is more uniform after beam shaping because the bottom of the flattened Gaussian beam is flat instead of a Gaussian curve. Therefore, compared to an ordinary Gaussian beam, the energy of a flattened Gaussian beam is more concentrated, which is favorable for the formation of a smaller heat-affected zone.

We quote the field distribution of a flattened Gaussian beam from F. Gori as follows [16]:

(1) $$\begin{array}{c} U_{N}\left(r,z\right)\\ =A\frac{v_{0N}}{v_{N\left(z\right)}}exp\left\{i\left[kz-\Phi_{N}\left(z\right)\right]\right\}\times exp\left[\left(\frac{ik}{2R_{N}\left(z\right)}-\frac{1}{v_{N\left(z\right)}^{2}}\right)r^{2}\right]\\ \times \;\;\;\;\;\;\;\;\;\overset{N}{\underset{n=0}{\sum }}c_{n}L_{n}(\frac{2r^{2}}{v_{N\left(z\right)}^{2}})exp\left[-2in\phi_{N}\left(z\right)\right] \end{array}$$

where

$v_{0N}=w_{0}/\sqrt{N}$, with $w_{0}$ is the waist radius at the plane *z* = 0,

$R_{N}\left(z\right)=z+z_{R}^{2}/z$,

$\Phi_{N}\left(z\right)=tan^{-1}\left(\frac{z}{z_{R}}\right)$,

$L_{n}$ is the *n*th Laguerre polynomial,

cn=(−1)n∑m=nN()nm12m,

k=2π/λ is the wavenumber (λ is the wavelength),

$v_{N\left(z\right)}=v_{0}\sqrt{1+{\left(\frac{z}{z_{R}}\right)}^{2}}$,

$z_{R}=kv_{0}^{2}/2$ ($z_{R}$ is called the Rayleigh distance),

*r* is the radial distance from the center axis of the beam,

*z* is the axial distance from the beam focus (or “waist”),

*i* is the imaginary unit,

$v_{0}$ is the spot size at the waist, and

N is the order of the flattened Gaussian beam.

**Figure 3:**
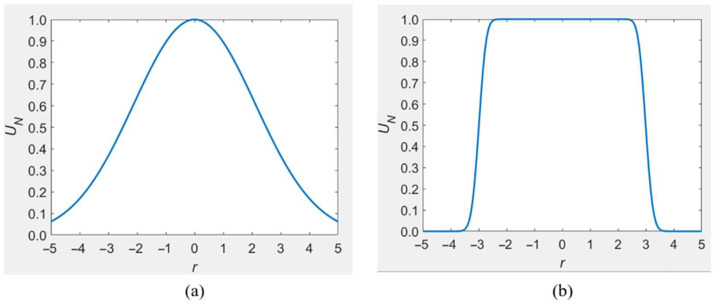
(**a**) Gaussian beam; (**b**) corresponding flattened Gaussian beam. ($w_{0}$ = 3 and (**a**) *N* = 0; (**b**) *N* = 50).

A comparison of a Gaussian beam and a flattened Gaussian beam is shown in Figure 3.

### 3.2. Formation of Multiple Refractive Indices

In fused silica, for high repetition rates, the time between femtosecond laser pluses is less than the time for heat to diffuse away, resulting in an accumulation of heat in the focal volume. Nonlinear effects such as self-focusing, plasma defocusing, and energy depletion influence the propagation of focused femtosecond laser pulses in glass, which alters the energy distribution at the focus and changes the refractive index [17,18,19]. Therefore, multiple refractive indices are formed in glass by periodically changing the repetition rate within the high repetition rate range.

### 3.3. Three Modification Physics

**Figure 4:**
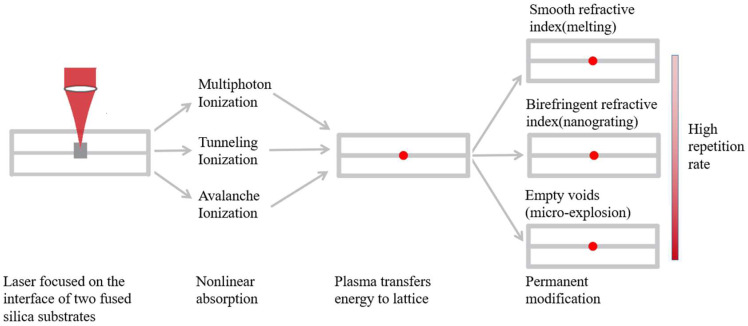
Three modification physics of femtosecond laser pulses interacting with fused silica [20].

Figure 4 illustrates the three-modification physics of femtosecond laser pulses interacting with fused silica. First, the laser beam is focused on the interface of two fused silica substrates. Then, nonlinear multiphoton ionization, tunneling ionization, and avalanche ionization from absorbed femtosecond laser pulses are responsible for the creation of a free electron plasma. Finally, the plasma transfers its energy to the lattice, resulting in three types of permanent modification: smooth refractive index, birefringent refractive index, and empty voids as the high repetition rate increases [20].

## 4. Results and Discussion

### 4.1. Results and Discussion of the Welding Spot Structure

**Figure 5:**
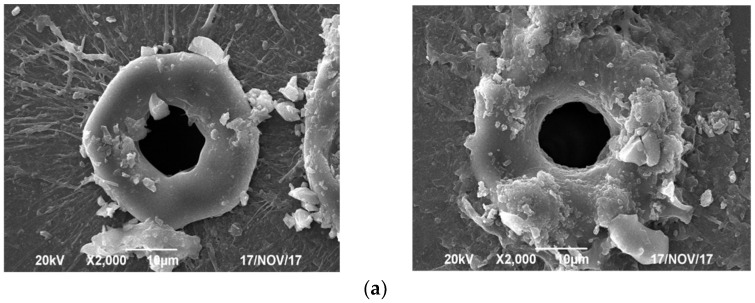

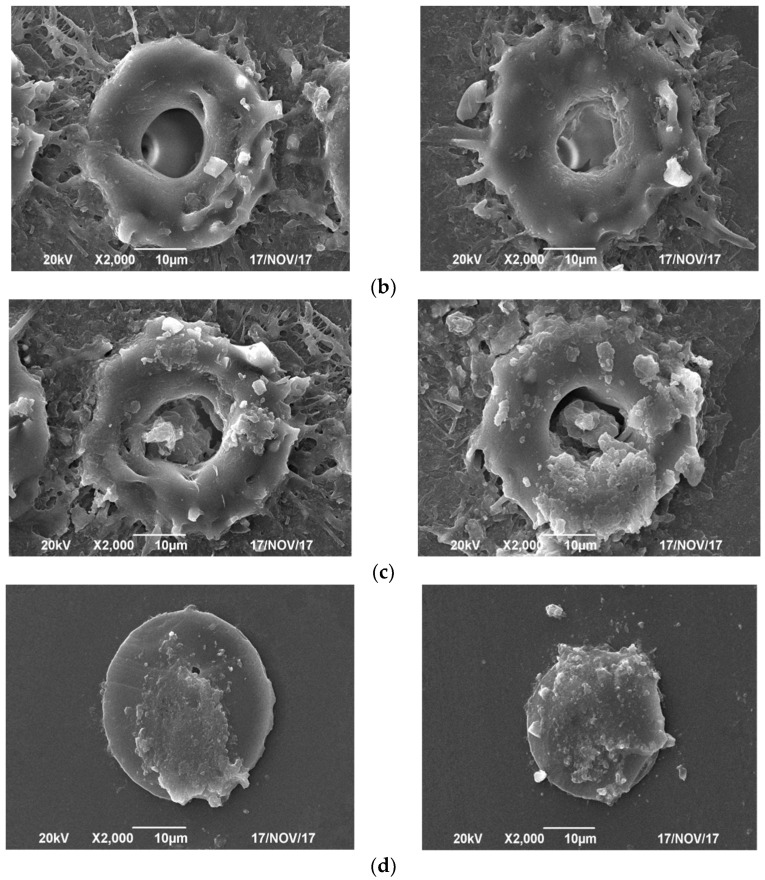
Structures of four types of welding spots. (**a**) Deep pores (depth: approximately 12 μm) (manufacturing processes: spatiotemporal beam shaping (flattened Gaussian beam); alternating high repetition rate transformation (600 kHz, 800 kHz and 1 MHz as one cycle)). (**b**) Partial deep pores (depth: approximately 5–12 μm) (manufacturing processes: spatiotemporal beam shaping (flattened Gaussian beam); high repetition rate set to 800 kHz). (**c**) Shallow pores (depth: approximately 5 μm) (manufacturing processes: no spatiotemporal beam shaping (Gaussian beam); alternating high repetition rate transformation (600 kHz, 800 kHz and 1 MHz as one cycle)). (**d**) No pores (manufacturing processes: no spatiotemporal beam shaping (Gaussian beam); high repetition rate set to 800 kHz).

Figure 5 shows the structure of four types of welding spots under a scanning electron microscope (SEM), 2000×. These welding spots were marked by the appearance of special porous structures by the use of spatiotemporal beam shaping, alternating high repetition rate transformation (the high repetition rate was periodically changed among 600 kHz, 800 kHz and 1 MHz as one cycle), both processes, or neither process. The corresponding welding spots presented detailed structures of deep pores, partial deep pores, shallow pores and no pores, as shown in Figure 5a–d.

As mentioned in Section 3.1, a Gaussian beam can be converted to a flattened Gaussian beam through spatiotemporal beam shaping. The corresponding flattened Gaussian beam possesses flat bottom characteristics. The energy is more uniformly distributed and concentrated on the focal position with little energy loss. Therefore, the laser energy is sufficient to drill deep pores and partial deep pores, as shown in Figure 5a,b, respectively. A heat-affected zone and oxidization are not apparent for the flattened Gaussian beam, which corresponds to the mechanism in Section 3.1. We can see that the deep pores and partial deep pores in Figure 5a,b have a fair quality of smoother welding edges with little debris. The bottom of the welding spot pore is flat.

However, comparing the deep pores in Figure 5a to the partial deep pores in Figure 5b, which both use spatiotemporal beam shaping, a possible reason for their difference is that the high repetition rate of 1 MHz as a key determining factor reaches the condition of the void formation mechanism, as previously described in Section 3.3. As a result, more fully deep pores are presented in Figure 5a. A similar phenomenon is proven once again in Figure 5c,d without spatiotemporal beam shaping, and the high repetition rate of 1 MHz may be beneficial to void formation.

As stated in Section 3.2, the heat built up at high repetition rates induces changes in the refractive index. If we periodically alternate the high repetition rate as in Figure 5a,c, then multiple refractive indices form in the welding spot pore.

**Figure 6:**
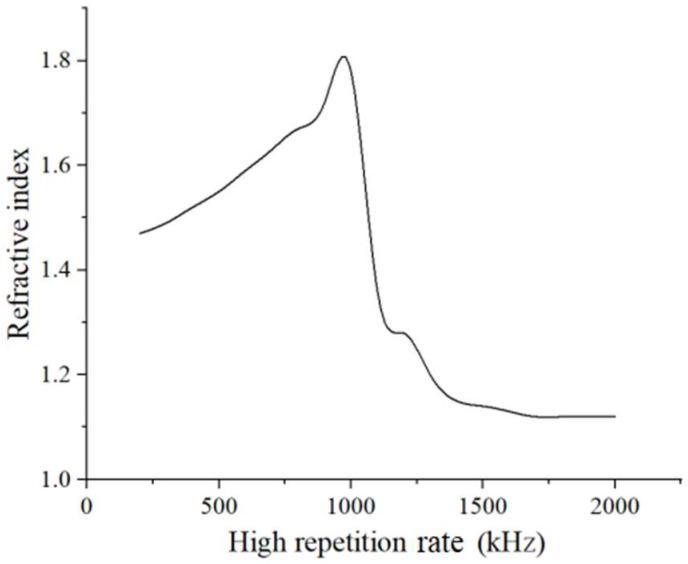
Relationship of the refractive index and high repetition rate (the original refractive index of JGS2 is 1.45).

Figure 6 illustrates the refractive index changes with the high repetition rate. The high repetition rate ranges from 200 kHz to 2 MHz. We can see that the refractive index rapidly grows with increasing high repetition rate from 200 kHz to 1 MHz. Above 1 MHz, the refractive index sharply decreases as the high repetition rate increases up to 1.7 MHz. The most likely cause of the sharp decline in the refractive index may be crack initiation and thermal stress generation due to excessive heat accumulation in the pore of the welding spot. Above 1.7 MHz, the curve becomes gentle as the high repetition rate increases to 2 MHz. A possible explanation for this is that cracks and thermal stress may reach a low threshold, and the refractive index will not decrease further. Consequently, high repetition rates from 200 kHz to 1 MHz are a proper range for manufacturing porous glass with multiple refractive indices in our experimental conditions. In the meantime, by measuring the bonding strengths of two welded glass substrates, the maximum value (71.3 MPa) appears at the high repetition rate of 1 MHz, which has obvious uniformity with the trends of refractive index.

By comparing Figure 5a–c or Figure 5b–d, we can tell that the pore formation is largely attributed to using spatiotemporal beam shaping rather than alternating high repetition rate transformation.

From what has been discussed above, we can make four conclusions. (1) In comparison to a Gaussian beam, a flattened Gaussian beam has greater drilling capacity. (2) The periodic change in the high repetition rate contributes to the formation of multiple refractive indices. (3) Higher repetition rates are favorite for void formation in the welding spot pore. (4) In comparison to alternating high repetition rate transformation, spatiotemporal beam shaping dominates the formation of pores.

### 4.2. Corresponding Functions of Porous Glass

According to the above results and discussion of manufacturing processes based on femtosecond laser welding, we can infer the potential functions and application prospects of porous glass as follows.

(1) The pores fabricated by a flattened Gaussian beam have a larger surface area, a deeper depth, a lower surface roughness, and a more regular shape. All of these properties are beneficial for manufacturing porous glass with an absorption or separation [21] function and the ability to serve as a catalyst carrier or photocatalyst carrier [22,23].

(2) Previous research demonstrated that the refractive index change induced by femtosecond laser irradiation of glass can be applied to optical storage [24,25] and structural color [26,27]. Higher storage capability and read efficiency can be obtained as the refractive index changes. Different colors can be observed in irradiated areas with different refractive indices of glass. Therefore, in our work, the multiple refractive indices obtained through alternating high repetition rate transformation provide favorable conditions for manufacturing porous glass with a multidimensional optical storage function or abundant structural color.

(3) In addition, empty voids created by microexplosions are a favorable structure for optical memory technology [28]. Thus, void formation is also conducive to manufacturing porous glass with the function of optical storage.

## 5. Conclusions

In this paper, we proposed two porous glass manufacturing processes based on femtosecond laser welding technology: spatiotemporal beam shaping and alternating high repetition rate transformation. Four kinds of welding spots were fabricated by a Gaussian beam or a flattened Gaussian beam with various high repetition rates. As a result, the welding spots exhibited different porous structures with multiple refractive indices. Compared to the traditional porous glass manufacturing methods, the processes have the advantages of one-time forming, no chemical material involved, a short production cycle, and a smaller heat-affected zone. The pores in welding spots formed through the above processes exhibit special structures and excellent properties, providing a prospective technology by which to manufacture functional porous glass. Additionally, reasonable selection of the high repetition rate range is a key factor in manufacturing functional porous glass. The porous glass manufacturing processes based on femtosecond laser welding may be further extended to other functional applications concerning super hydrophobicity, luminescence, sound absorption, etc. in our future work.

## References

[1] Janowski F., Enke D. (2002). Porous Glasses. Handbook of Porous Solids.

[2] Hood H.P., Nordberg M.E. (1942). Method of Treating Borosilicate Glasses.

[3] Rabinovich E. (1985). Preparation of glass by sintering. J. Mater. Sci. Rev..

[4] Sacks M.D., Tseng T.-Y. (1984). Preparation of SiO_2_ Glass from Model Powder Compacts: II, Sintering. J. Am. Ceram. Soc..

[5] Sadighzadeh A., Ghoranneviss M., Elahi A.S. (2014). Application of partial sintering of waste glasses for preparation of porous glass bodies. J. Porous Mater..

[6] Yazawa T., Tanaka H., Eguchi K. (1994). Preparation of porous glass hollow fibre from glass based on SiO_2_-B_2_O_3_-RO-ZrO_2_ (R = Ca, Zn) system. J. Mater. Sci. Lett..

[7] Kokubu T., Yamane M. (1985). Preparation of porous glass-ceramics of the TiO_2_-SiO_2_ system. J. Mater. Sci..

[8] Hammel J.J., Allersma T. (1976). Thermally Stable and Crush Resistant Microporous Glass Catalyst Supports and Methods of Making.

[9] Santos A.M.M., Vasconcelos W.L. (2000). Properties of porous silica glasses prepared via sol–gel process. J. Non-Cryst. Solids.

[10] Gonzalez-Oliver C.J.R., James P.F., Rawson H. (1982). Silica and silica-titania glasses prepared by the sol-gel process. J. Non-Cryst. Solids.

[11] Awschalom D., Warnock J. (1987). Supercooled liquids and solids in porous glass. Phys. Rev. B.

[12] Zhang H., Eaton S.M., Li J., Nejadmalayeri A.H., Herman P.R. (2007). Type II high-strength Bragg grating waveguides photowritten with ultrashort laser pulses. Opt. Express.

[13] Zhang F., Xie X., Zhao X., Ma L., Lei L., Qiu J., Nie Z. (2021). Polarization-dependent microstructural evolution induced by a femtosecond laser in an aluminosilicate glass. Opt. Express.

[14] Jiang L., Liu P., Yan X., Leng N., Xu C., Xiao H., Lu Y. (2012). High-throughput rear-surface drilling of microchannels in glass based on electron dynamics control using femtosecond pulse trains. Opt. Lett..

[15] Duerr F., Thienpont H. (2014). Refractive laser beam shaping by means of a functional differential equation based design approach. Opt. Express.

[16] Gori F. (1994). Flattened gaussian beams. Opt. Commun..

[17] Homoelle D., Wielandy S., Gaeta A.L., Borrelli N.F., Smith C. (1999). Infrared photosensitivity in silica glasses exposed to femtosecond laser pulses. Opt. Lett..

[18] Will M., Nolte S., Chichkov B.N., Tünnermann A. (2002). Optical properties of waveguides fabricated in fused silica by femtosecond laser pulses. Appl. Opt..

[19] Osellame R., Chiodo N., Valle G.D., Cerullo G., Ramponi R., Laporta P., Killi A., Morgner U., Svelto O. (2006). Waveguide lasers in the C-band fabricated by laser inscription with a compact femtosecond oscillator. IEEE J. Sel. Top. Quantum Electron..

[20] Itoh K., Watanabe W., Nolte S., Schaffer C.B. (2006). Ultrafast Processes for Bulk Modification of Transparent Materials. MRS Bull..

[21] Sun M.-H., Huang S.-Z., Chen L.-H., Li Y., Yang X.-Y., Yuan Z.-Y., Su B.-L. (2016). Applications of hierarchically structured porous materials from energy storage and conversion, catalysis, photocatalysis, adsorption, separation, and sensing to biomedicine. Chem. Soc. Rev..

[22] Takasu Y., Kawaguchi T., Sugimoto W., Murakami Y. (2003). Effects of the surface area of carbon support on the characteristics of highly-dispersed Pt-Ru particles as catalysts for methanol oxidation. Electrochim. Acta.

[23] Takahashi T., Iwaishi S.-I., Yanagimoto Y., Kai T. (1997). Hydrogenation of 1-hexenes and 1-octenes over nickel catalyst supported on porous glass prepared from borosilicate glass. Korean J. Chem. Eng..

[24] Chen D., Zook J.D. (1975). An overview of optical data storage technology. Proc. IEEE.

[25] Qiu J., Miura K., Hirao K. (1998). Three-dimensional optical memory using glasses as a recording medium through a multi-photon absorption process. Jpn. J. Appl. Phys..

[26] Takeoka Y. (2012). Angle-independent structural coloured amorphous arrays. J. Mater. Chem..

[27] Luo Y., Zhang J., Sun A., Chu C., Zhou S., Guo J., Chen T., Xu G. (2014). Electric field induced structural color changes of SiO_2_@ TiO_2_ core–shell colloidal suspensions. J. Mater. Chem. C.

[28] Glezer E., Milosavljevic M., Huang L., Finlay R., Her T.-H., Callan J.P., Mazur E. (1996). Three-dimensional optical storage inside transparent materials. Opt. Lett..

